# NIT1 suppresses tumour proliferation by activating the TGFβ1–Smad2/3 signalling pathway in colorectal cancer

**DOI:** 10.1038/s41419-018-0333-3

**Published:** 2018-02-15

**Authors:** Chun Lin, Jianming Zhang, Yanxia Lu, Xiaomin Li, Wenjuan Zhang, Wei Zhang, Weihao Lin, Lin Zheng, Xuenong Li

**Affiliations:** 1 Department of Pathology, School of Basic Medical Sciences, Southern Medical University, Guangzhou, China; 2 Department of Surgery, Nanfang Hospital, Southern Medical University, Guangzhou, China

## Abstract

NIT1 protein has been reported to be a potential tumour suppressor in tumour progression. However, little is known about the specific role of NIT1 in tumour development and progression. In this study, we confirmed the specific effects of NIT1 in the regulation of colorectal carcinoma cell proliferation. Here, we showed that NIT1 was significantly downregulated in colorectal cancer tissues compared with that in adjacent normal tissues. The decreased expression of NIT1 was significantly correlated with poor differentiation and more serosal invasion. Functional experiments showed that NIT1 inhibited CRC cell growth both in vitro and in vivo. NIT1 induced cell cycle arrest and apoptosis. Furthermore, NIT1 recruited Smad2/3 to the TGFβ receptor and activated the TGFβ–Smad2/3 pathway by interacting with SARA and SMAD2/3 in CRC. Further study has shown that SMAD3 directly binds to the promoter regions of NIT1 and enhances the transcription of NIT1. Together, our findings indicate that NIT1 suppresses CRC proliferation through a positive feedback loop between NIT1 and activation of the TGFβ–Smad signalling pathway. This study might provide a new promising strategy for CRC.

## Introduction

Colorectal cancer (CRC) is the third most common cancer in men and second most common cancer in women worldwide (http://globocan.iarc.fr)^1^. CRC is a high-risk, digestive-tract malignant tumour with a high prevalence and remains one of the leading causes of cancer mortality. Although the diagnosis and treatment of CRC have made significant progress in the last ten years, the incidence and mortality will also remain high long term^2,3^. The occurrence and development of CRC is a complex procedure resulting from multiple genetic and epigenetic changes that lead to a multistep and stepwise progression from normal mucosa to dysplasia and finally to carcinoma. Tumourigenesis generally arises as a consequence of the activation of oncogenes or inactivation of tumour suppressor genes^4^. For example, the inactivating mutation of adenomatous polyposis coli plays an important role in colorectal tumourigenesis via the aberrant regulation of intracellular β-catenin to activate the Wnt pathway^5^. TP53 mutation leads to inactivation of the p53 pathway and regulation of cell cycle and apoptosis^6^. However, the detailed molecular mechanisms of CRC remain unclear.

Transforming growth factor β (TGFβ) super family signalling plays a key role in the regulation of cell proliferation, differentiation, apoptosis, and development in numerous biological systems^7^. In general, signalling starts with ligand-induced oligomerization of serine/threonine receptor kinases and phosphorylation of the cytoplasmic signalling molecules Smad2 and Smad3 for the TGFβ pathway. Smads function with the common signalling transducer Smad4 to be co-transported to the nucleus after C-terminal phosphorylation by activated receptors^7–9^. Activated Smads modulate various biological effects by partnering with other transcription factors that lead to cell-state-specific regulation of transcription^10^. ZFYVE (SARA) participates in the TGFβ signalling pathway by directly interacting with SMAD2 and SMAD3, and recruiting them to the TGFβ receptor^10,11^.

The protein coding gene human nitrilase 1 (NIT1), a member of the carbon-nitrogen hydrolase superfamily with homology to bacterial and plant nitrilases, enzymes that cleave nitriles and organic amides to the corresponding carboxylic acids plus ammonia^12–14^. In *Drosophila melanogaster* and *Caenorhabditis elegans*, NitFhit protein is a fusion protein composed of a C-terminal fhit field and an area related to plant and bacterial hydrolysis^15^. In humans and mice, the nitrilase homologues and Fhit were encoded by two different genes, FHIT and NIT1, located on chromosomes 3 and 1 in humans, and 14 and 1 in mice, respectively. Based on the Rosetta Stone theory, the presence of a fusion protein in a genome predicts that the isolated polypeptides function in the same cellular or biochemical pathways in other organisms^16–19^. It is generally known that FHIT is a clear tumour suppressor gene that suppresses cell growth and promotes apoptosis. Loss of FHIT is associated with many human malignancies^20–22^. Knockdown of NIT1 promotes cell proliferation, clonogenicity and resistance to DNA damage stress in mouse kidney cells. Conversely, overexpression of NIT1 suppressed cell viability and induced caspase-dependent apoptosis^23^. The deficiency of NIT1 resulted in the increased incidence of *N-*nitrosomethylbenzylamine-induced murine forestomach tumours^23^. The overexpression of NIT2, another nitrilase superfamily member that shares 55% homology to NIT1, suppressed cell growth and induced G2 arrest in HeLa cells^24^. The above evidence have suggested that NIT1 might be a potential tumour suppressor candidate. However, its definite biological functions and regulatory mechanisms in human carcinoma including CRC remain elusive.

In this study, we aim to investigate the potential role of NIT1 in the development and progression of CRC. Here, we found that NIT1 is significantly downregulated in CRC tissues compared with that in adjacent normal tissues. Additionally, the downregulation of NIT1 is correlated with CRC progression and a poor prognosis. Moreover, we found that NIT1 inhibits CRC cell proliferation by interacting with SARA to recruit Smad2/3 to the TGFβ receptor to activate the TGFβ–Smad pathway in CRC. Furthermore, NIT1 induces cell cycle arrest and caspase3-dependent apoptosis. In addition, we showed that SMAD3 could directly bind to the promoter regions of NIT1 and enhance the transcription of NIT1. Our study provides novel insight into the function and mechanism of NIT1 in CRC pathogenesis.

## Results

### Downregulation of NIT1 is correlated with CRC progression and poor prognosis

**Fig. 1:**
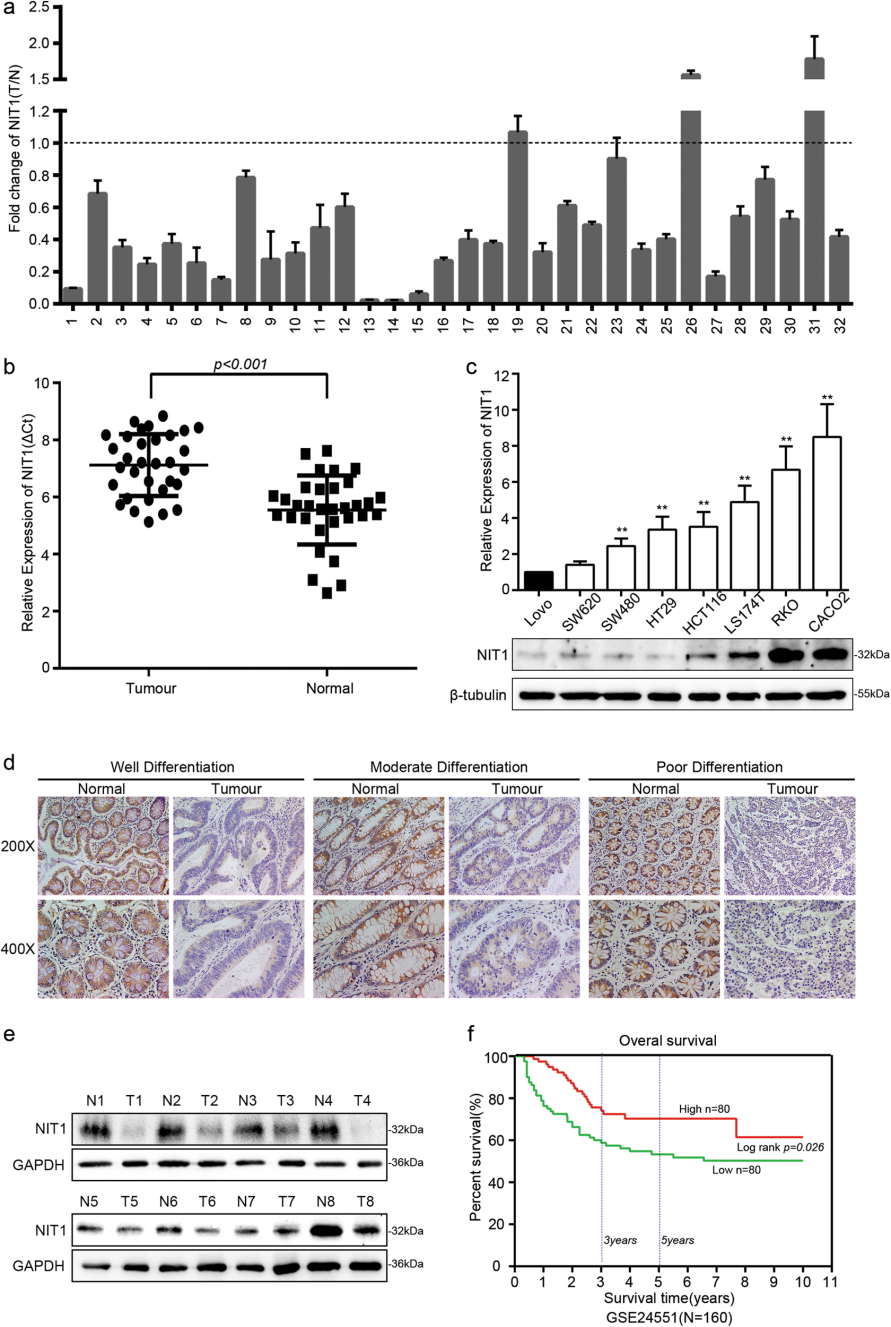
Downregulation of NIT1 is correlated with CRC progression and poor prognosis. **a** qRT-PCR analysis of NIT1 expression in 32 paired human colorectal cancer tissues. NIT1 was quantified relative to matched adjacent normal tissues and was normalized to GAPDH. Error bars represent the means ± SD of three independent experiments. **b** Comparison of NIT1 expression in 32 paired CRC tissues (T) using matched adjacent normal tissues (N) (*p* < 0.001). **c** qRT-PCR and western blot analysis of NIT1 expression in eight CRC cell lines. Error bars indicate the means ± SD of three parallel experiments (**p < 0.01). **d** Immunohistochemical staining (IHC) of NIT1 protein in CRC tissues and normal intestinal epithelium tissues. Representative images as shown above. **e** Western blotting of NIT1 expression in eight fresh surgically resected human CRC tissues (T) and paired adjacent normal intestine epithelial samples (N). **f** Influence of NIT1 expression on overall survival using Kaplan–Meier analysis in 160 CRC patients from a public clinical microarray dataset of GSE24551

We relied on Oncomine, a cancer microarray database and web-based data-mining platform, to identify the expression level of NIT1 in CRC tissues, the results indicated that NIT1 was significantly downregulated in CRC tissues compared with that in adjacent normal tissues (*p* < 0.001) (Supplementary Figure S1). We analysed the mRNA expression levels of NIT1 in 32 paired CRC tissues and paired normal tissues using quantitative reverse transcription PCR (qRT-PCR). The average expression level of NIT1 was significantly decreased in 29 of 32 CRC specimens compared with their adjacent normal mucosa tissues (*p* < 0.001) (Fig. 1a, b). Consistently, NIT1 was downregulated in CRC specimens compared with that in their adjacent normal mucosa tissues by western blotting analysis (Fig. 1e). We also evaluated the expression levels of NIT1 in eight CRC cell lines, including Lovo, SW620, SW480, HT29, HCT116, LS174T, RKO and CACO2, using qRT-PCR and western blotting analysis. Relative NIT1 expression was significantly lower in highly metastatic CRC cell lines SW620 and Lovo than in the low metastasis cell lines SW480, HT29, HCT116, LS174T, RKO and CACO2 (Fig. 1c). We further detected the expression level of NIT1 in 69 cases of paraffin-embedded CRC tissue sections using immunohistochemical (IHC) assays. NIT1 is located predominantly in the cytoplasm. Distinctly decreased expression of NIT1 was observed in 84% (58/69) of CRC tumour tissues compared with that in adjacent normal tissues (Fig. 1d). The expression level of NIT1 was divided into a high-expression group (*n* = 19) and a low-expression group (*n* = 50) to investigate the relationship between NIT1 expression levels and the clinicopathological characteristics of tumour tissue samples. As summarized in Table 1, the low expression level of NIT1 protein was significantly correlated with poor differentiation (*p* < 0.001) and more serosal invasion (*p* < 0.001). The results of Kaplan–Meier survival analysis also indicated that patients with low NIT1 expression levels had a poor prognosis in 160 CRC patients from a public clinical microarray database of GSE24551 (Fig. 1f).

**Table 1:** Clinicopathologic characteristics of NIT1 expression in CRC patients

Clinicopathological variables	*N*	High expression	Low expression	*X* ^2^	*P*
All cases	69	19	50		
Age(years)					
≤60	31	6	25	1.888	0.169
>60	38	13	25		
Gender					
Male	34	9	25	0.038	0.845
Female	35	10	25		
Tumour size (cm)					
≤4.75	37	11	26	0.192	0.661
>4.75	32	8	24		
Differentiation					
Well	13	10	3	21.693	0.000
Moderate	46	9	37		
Poor	10	0	10		
Serosal invasion					
Yes	45	3	42	28.239	0.000
No	24	16	8		
Lymph metastasis					
Yes	37	10	27	0.01	0.919
No	32	9	23		
TNM classification					
I–II	43	13	30	0.416	0.519
III–IV	26	6	20		

### NIT1 inhibits CRC cell proliferation in vitro and tumour growth in vivo

**Fig. 2:**
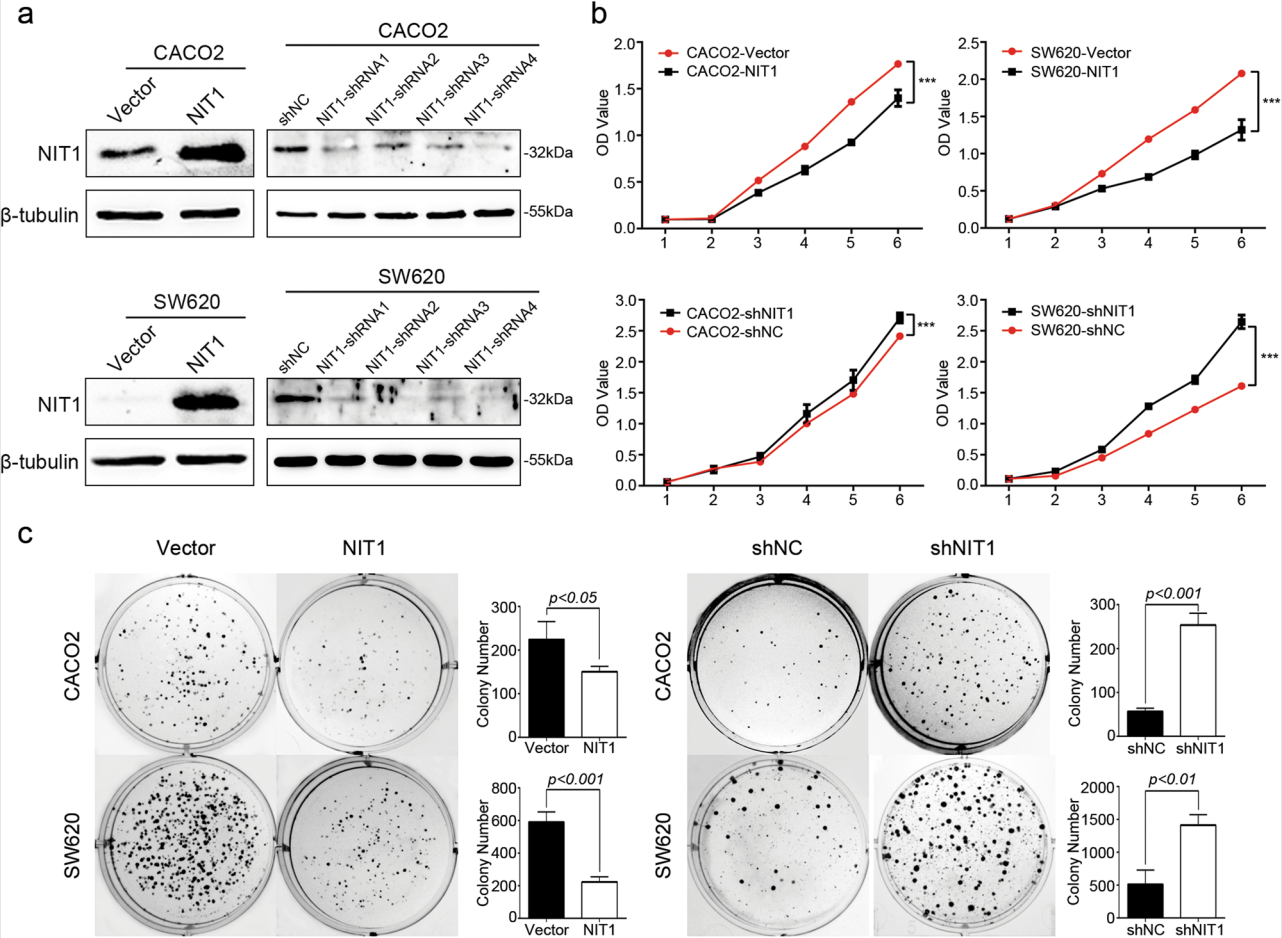
NIT1 inhibits CRC cell proliferation in vitro. **a** Overexpression or knockdown of NIT1 in CACO2 and SW620 cells analysed by western blotting. β-tubulin was used as a loading control. **b** Effects of NIT1 on cell proliferation were determined by CCK-8 cell proliferation assays. The data are represented as the means ± SD of five samples. **c** Effects of NIT1 on cell proliferation were determined by colony formation assays. The data are represented as the means ± SD of three samples

**Fig. 3:**
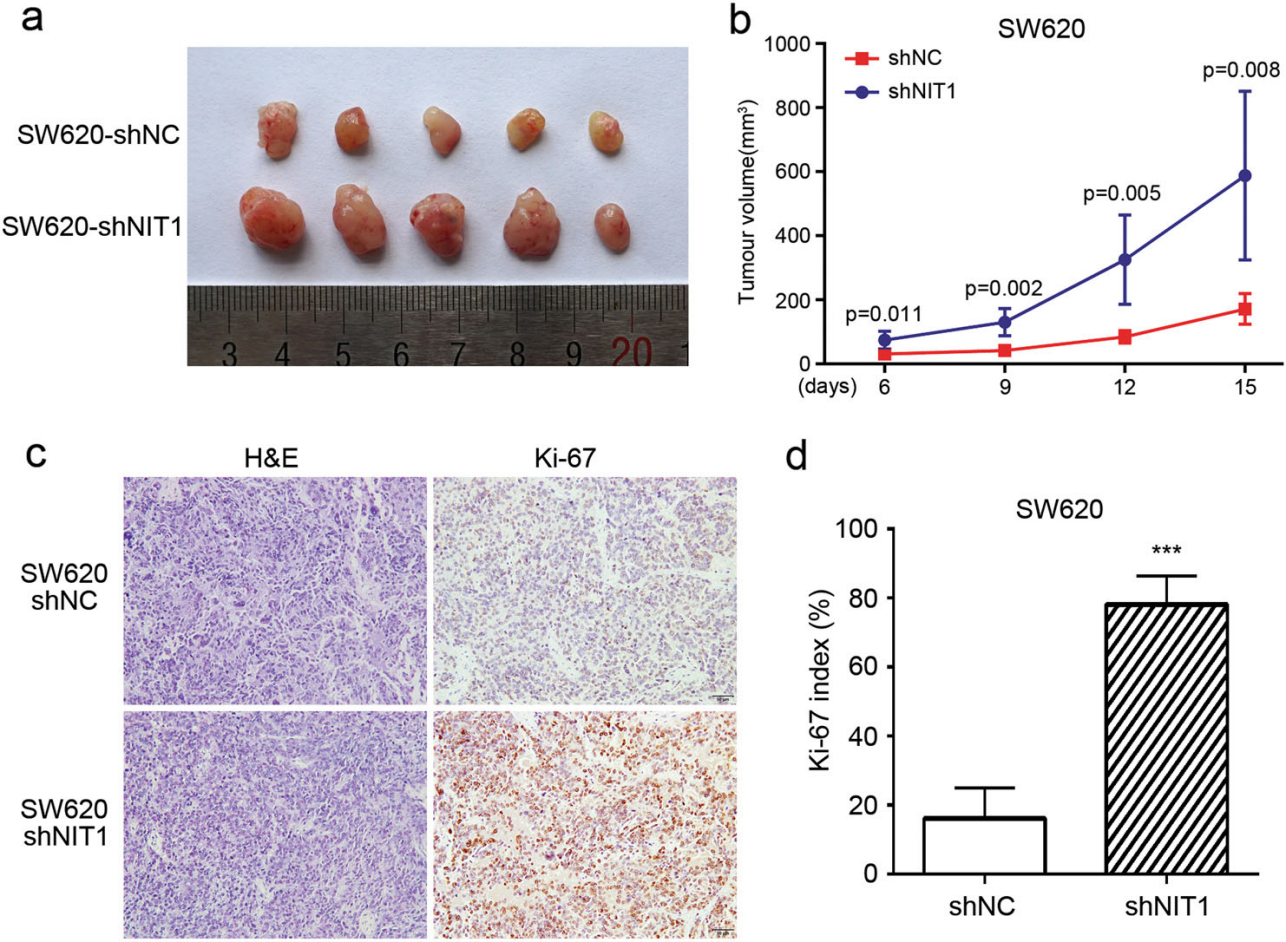
Knockdown of NIT1 promotes tumour growth in vivo. **a** SW620-shNC cells and SW620-shNIT1 cells were subcutaneously injected into nude mice (*n* = 5), and xenograft models were generated. **b** The tumour sizes were measured on the indicated days. The data are represented as the mean tumour volumes ± SD for five samples. **c** Sections of subcutaneous tumours were subjected to H&E and IHC staining using an antibody against Ki-67. **d** The Ki-67 index was calculated as the number of Ki-67-positive cells divided by the number of total cells × 100% (magnification, ×200). Error bars represent the means ± SD of five different fields

To evaluate the possible role of NIT1 in CRC proliferation, the stable NIT1 expressed cell lines CACO2-NIT1 and SW620-NIT1 were established (Fig. 2a). We also knocked down endogenous NIT1 using four independent NIT1-shRNAs in CACO2 and SW620 cells (Fig. 2a). NIT1-shRNA4 was used to establish CRC cell lines constitutively repressing NIT1 to further confirm the impact of NIT1 on CRC cell proliferation (Fig. 2a). The results of CCK-8 cell proliferation assays revealed that the over-expression of NIT1 significantly restrained the cell proliferation of CACO2 and SW620 cells compared with that of their control cells (Fig. 2b; *p* < 0.001). By contrast, knockdown of NIT1 in CACO2 and SW620 yielded opposite effects (Fig. 2b; *p* < 0.001). Plate colony formation assays revealed that CACO2-NIT1 and SW620-NIT1 cells formed fewer and smaller colonies than control cells (Fig. 2c; *p* < 0.05). However, the silencing of NIT1 obviously promoted the colony formation viability of CACO2 (*p* < 0.001) and SW620 (*p* < 0.01) cells (Fig. 2c). We also performed CCK-8 cell proliferation assays and plate colony formation assays with NIT1-shRNA1 and NIT1-shRNA3 in CACO2 and SW620 cell lines, respectively. Our data were consistent with our previous results (Supplementary Figure S2; *p* < 0.05). Subsequently, we assessed the effect of NIT1 on tumour growth in a nude mouse xenograft model. SW620-shNC and SW620-shNIT1 cells were subcutaneously injected into nude mice, and the tumour size was measured over time. SW620-shNIT1 cells inoculated into nude mice developed larger and more quickly growing tumours compared with those in animals injected with SW620-shNC cells (Fig. 3a, b; *n* = 5; *p *< 0.01). Additionally, IHC staining of Ki-67 substantiated that the tumours formed in the SW620-shNIT1 groups displayed a higher Ki-67 proliferation index than those in the SW620-shNC group (Fig. 3c, d; *p* < 0.001).

### NIT1 induces cell cycle arrest and apoptosis

**Fig. 4:**
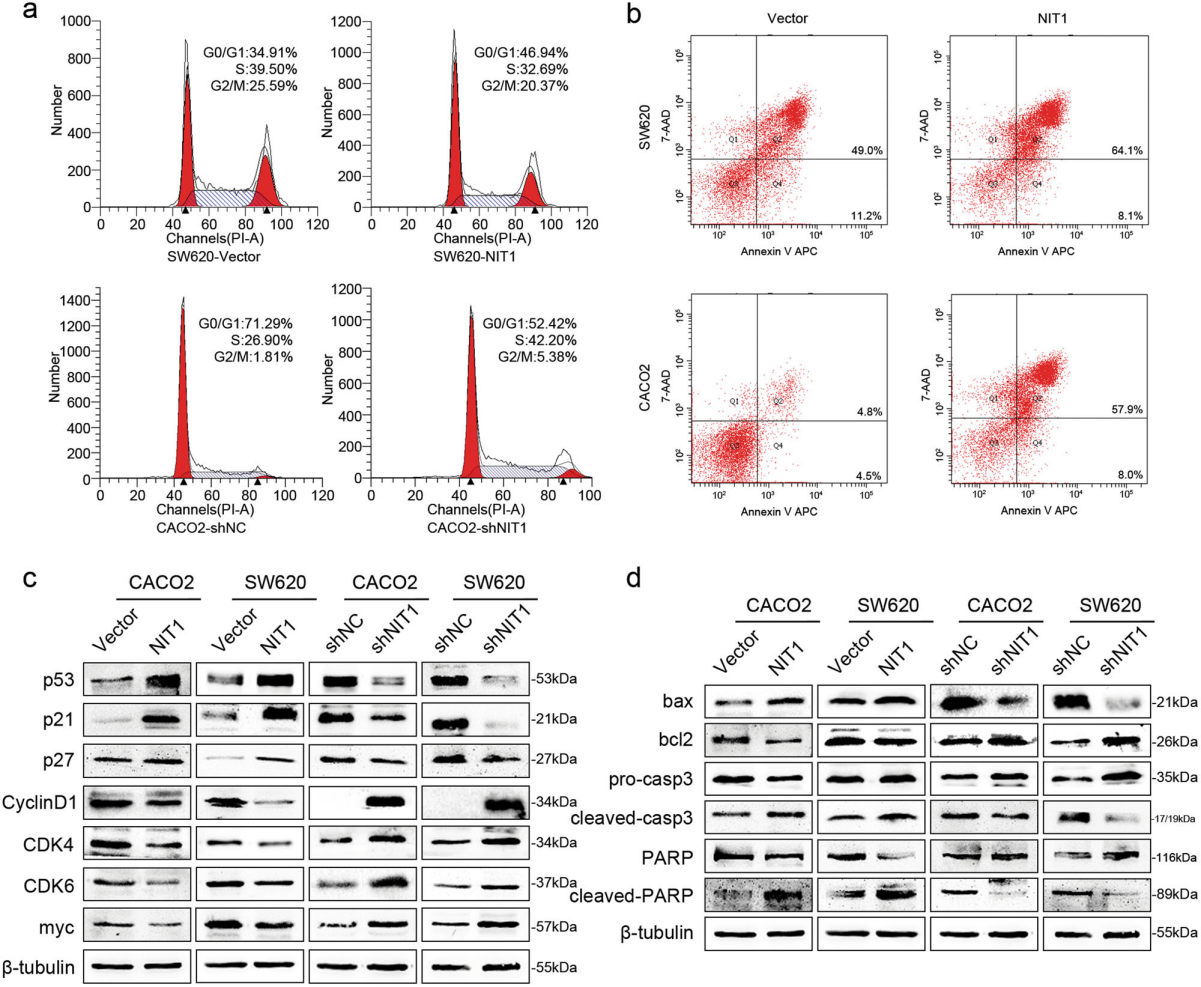
NIT1 induces cell cycle arrest and apoptosis. **a** The cell cycle distribution was analysed after the overexpression or knockdown of NIT1 by flow cytometry in CRC cells. **b** Overexpression of NIT1 promoted apoptosis as analysed by flow cytometry in CRC cells. **c** The expression levels of cell cycle regulators, including p53, p21, p27, CyclinD1, CDK4, CDK6 and myc, were analysed by western blot assays. **d** The expression of apoptosis-associated proteins, including bax, bcl2, caspase3 and PARP, were analysed by western blot assays

The results of gene set enrichment analysis revealed that the cell cycle-related signalling pathway was activated, and the apoptosis-related signalling pathway was suppressed when NIT1 was downregulated (Supplementary Figure S3). As shown in Fig. 4a, the overexpression of NIT1 in SW620 cells significantly increased the proportion of cells in G0/G1 phase (from 34.91 to 46.94%) but decreased the proportion of cells in S phase (from 39.50 to 32.69%). Conversely, the downregulation of NIT1 in CACO2 cells significantly decreased the proportion of cells in G0/G1 phase (from 71.29 to 52.42%) but increased the proportion of cells in S phase (from 26.90 to 42.20%). To better understand the underlying mechanisms that inhibit the G1 to S-cell cycle transition mediated by NIT1, the expression levels of some of the cell cycle regulators, including p53, p21, p27, CyclinD1, CDK4, CDK6 and myc, was detected. As shown in Fig. 4c, the overexpression of NIT1 induced significant upregulation of p53, p21 and p27, whereas the expression of CyclinD1, CDK4, CDK6 and myc was downregulated. By contrast, evident increases in the expression of CyclinD1, CDK4, CDK6 and myc and distinct decreases in the expression of p53, p21 and p27 were observed in NIT1-suppressed cells (Fig. 4c). The upregulation of NIT1 induced a significant increase in the total apoptosis rate in SW620 cells (60.2–72.2%) and CACO2 cells (9.3–65.9%) (Fig. 4b). Simultaneously, western blotting was used to detect the expression levels of some apoptosis-associated proteins, including bax, bcl2, caspase3 and PARP. The results revealed that NIT1 could activate caspase3 activity, which plays a crucial role in apoptosis, and then disintegrate the substrate PARP into cleaved PARP^25,26^. As shown in Fig. 4d, the upregulation of NIT1 increased the expression levels of cleaved caspase3 and cleaved PARP. The precursors of caspase3 and PARP remained unchanged or decreased. Additionally, the overexpression of NIT1 increased the expression of bax and decreased the expression of bcl2. Opposite effects appeared after knocking down the expression of NIT1 (Fig. 4d). Similar results were found in CRC cell lines using NIT1-shRNA1 and NIT1-shRNA3 (Supplementary Figure S4).

### NIT1 recruits Smad2/3 and then activates the TGFβ–Smad2/3 pathway by interacting with SARA and SMAD2/3 in CRC

**Fig. 5:**
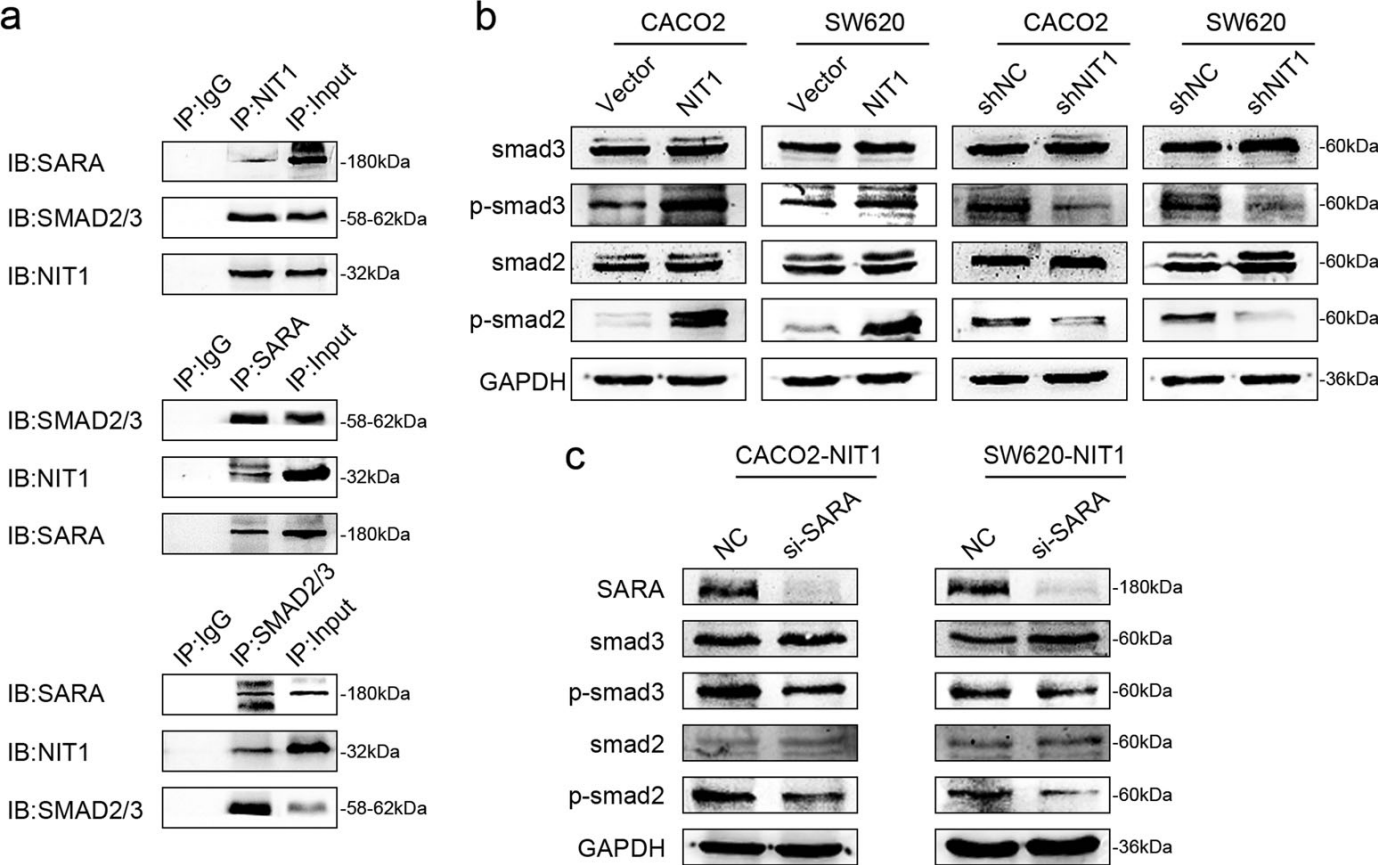
NIT1 recruits Smad2/3 and then activates the TGFβ-Smad2/3 pathway by interacting with SARA and SMAD2/3 in CRC. **a** Protein interactions among NIT1, SARA and SMAD2/3 using coimmunoprecipitation (co-IP) assays. **b** Western blot analysis of Smad3, p-Smad3, Smad2 and p-Smad2 after increasing or decreasing the expression of NIT1 in CRC cells as indicated. GAPDH served as the loading control. **c** Negative control or si-SARA was transfected into over-expressed NIT1 CRC cells to detect the expression levels of SARA, Smad3, p-Smad3, Smad2 and p-Smad2 using western blot assays. GAPDH served as the loading control

The molecular mechanisms underlying NIT1 in cell proliferation remain poorly identified. While analysing the public databases BioGRID (http://www.thebiogrid.org/), HPRD (http://www.hprd.org/), MINT (http://mint.bio.uniroma2.it/mint/Welcome.do), IntAct (https://www.ebi.ac.uk/intact/) and PrePPI (https://bhapp.c2b2.columbia.edu/PrePPI/), we noted that the protein NIT1 most likely interacted with ZFYVE9 (SARA/SMADIP). Previous studies indicated that the SARA gene encodes a double zinc finger motif-containing protein that participates in the TGFβ signalling pathway and suppresses tumour growth. SARA interacts directly with SMAD2 and SMAD3 and recruits SMAD2 and SMAD3 to the TGFβ receptor^10,11^. In the present work, we performed co-immunoprecipitation (co-IP) analysis and revealed that NIT1, SARA and SMAD2/3 interacted with each other (Fig. 5a). Thus, we hypothesized that NIT1 regulates CRC cell proliferation through the TGFβ–Smad2/3 signalling pathway. The upregulation of NIT1 evidently increased the phosphorylation levels of Smad2 and Smad3 (p-smad2/p-smad3) compared with those of their control cells. Conversely, the downregulation of NIT1 led to opposite effects. No change in the total protein amounts of Smad2 and Smad3 was observed under any circumstance (Fig. 5b). We also assessed whether NIT1 interacts with SARA to activate the TGFβ–Smad2/3 signalling pathway to modulate CRC proliferation. Transfection with the SARA-siRNA (si-SARA) construct in NIT1-overexpressing cells attenuated the phosphorylation of Smad2 and Smad3 (p-smad2/p-smad3) (Fig. 5c). Briefly, the TGFβ–Smad2/3 signalling pathway has been considered in relation to the maintenance of CRC growth inhibition. Additionally, activation of the TGFβ–Smad2/3 pathway by NIT1 might depend on the interaction with SARA.

### SMAD3 directly binds to the promoter regions of NIT1 and then enhances the transcription of NIT1

**Fig. 6:**
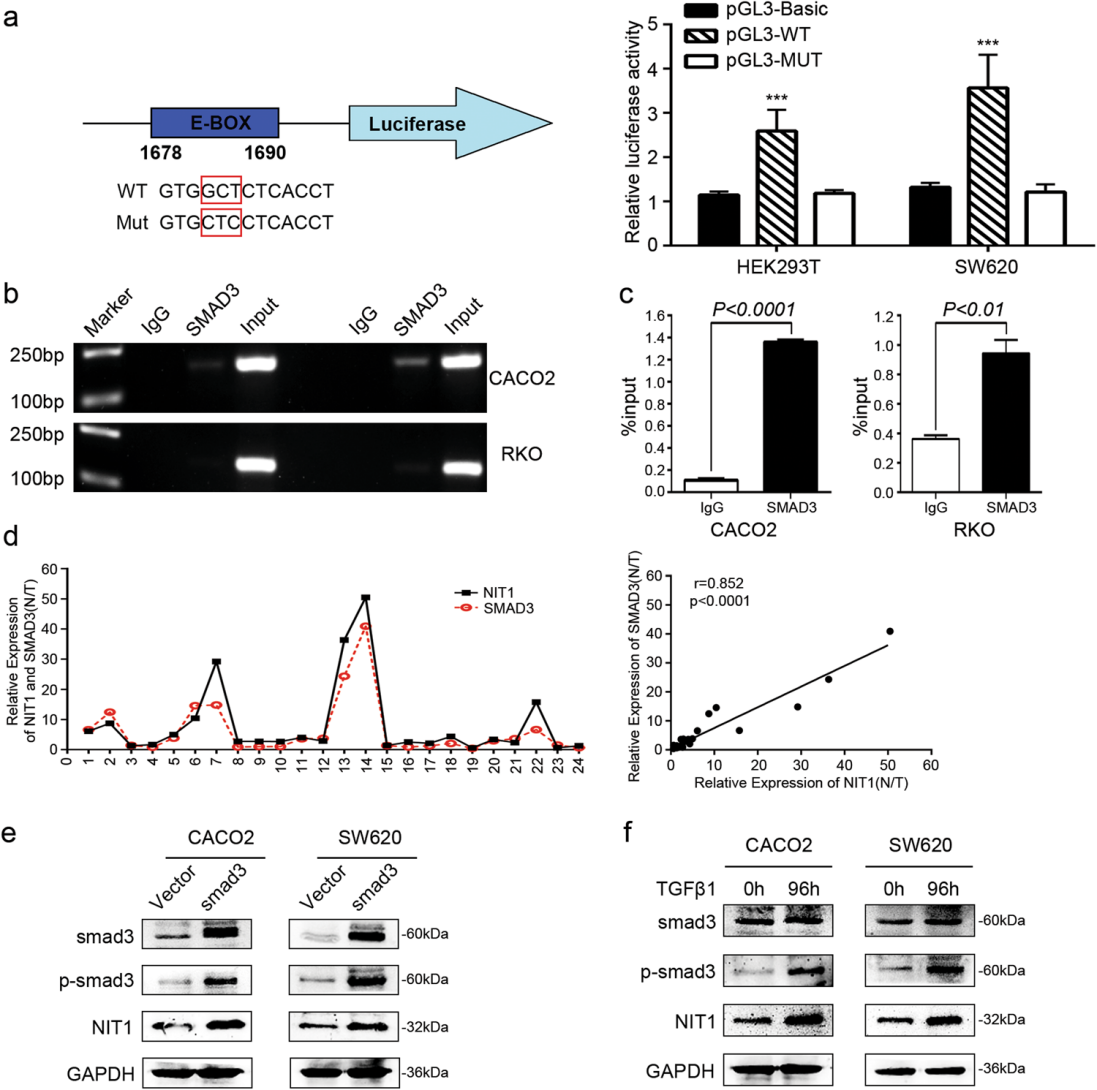
SMAD3 directly binds to the promoter regions of NIT1 and then enhances the transcription of NIT1. **a** A schematic of the NIT1 promoter luciferase reporter vector construct is depicted with the locations of the E-box element and sequences of the mutants. Relative luciferase activities of the WT or Mut E-box region in HEK293T and SW620 cells were detected after co-transfecting with the SMAD3 plasmid 72 h later. Values are the means ± SD of three independent assays. ***p < 0.001. **b** ChIP-PCR assays were used to assess SMAD3 binding at the promoter region of NIT1 containing the E-box element. **c** ChIP-qPCR assays were used to further confirm the SMAD3 binding at the promoter region of NIT1 containing the E-box element. The data indicate the means ± SD of three independent experiments. **d** qRT-PCR analysis of the expression of SMAD3 and NIT1 in 24 matched human colorectal cancer tissues and adjacent normal tissues. The expression levels of SMAD3 and NIT1 were normalized to those of GAPDH, and the results were presented as the fold change in paired adjacent normal tissues (N) relative to the tumour tissues (T). Spearman correlation analysis displayed a positive relationship between NIT1 and SMAD3 in the mRNA expression level of 24 paired CRC tissues (*r* = 0.852; *p* < 0.0001). **e** Western blotting analysis of the expression levels of NIT1 after transfecting the SMAD3 plasmid or the control vector in CRC cells. GAPDH served as the loading control. **f** Western blot assays were used to detect the expression levels of Smad3, p-Smad3 and NIT1 under the stimulation of TGFβ1 after 96 h

To examine the transcriptional regulation of NIT1, we utilized bioinformatics. While analysing the transcription start site (TSS) of NIT1 using the JASPAR database (http://jaspar.binf.ku.dk/), we found five SMAD2::SMAD3::SMAD4 transcription factor binding sites located within the NIT1 promoter (Supplementary Figure S5). Previous studies have reported that SMAD3, but not SMAD2, can act as a transcription factor by binding to a TGFβ-responsive sequence termed the CAGA box. SMAD2 differs from SMAD3 mainly in the N-terminal MH1 domain, which contains two additional stretches of amino acids that are lacking in SMAD3. Additionally, this domain prevents SMAD2 from binding to the DNA sequence^27–29^. Thus, the luciferase reporter assay was performed to detect the luciferase activity of the NIT1 promoter when SMAD3 was overexpressed. An increase in the wild-type (WT) NIT1 promoter luciferase activity was observed following the upregulation of SMAD3 in the HEK293T and SW620 cell lines (Fig. 6a). Additionally, chromatin immunoprecipitation (ChIP)-PCR and ChIP-qRT-PCR assays both confirmed that SMAD3 directly binds to specific regions (1678–1690 bp) of the NIT1 promoter in CACO2 and RKO cells (Fig. 6b, c, and Supplementary Figure S6). The upregulation of SMAD3 significantly increased the expression of NIT1 in CACO2 and SW620 cells (Fig. 6e). Spearman correlation analyses demonstrated that NIT1 and SMAD3 were positively correlated in expression (Fig. 6d; *r* = 0.852; *p* < 0.0001). Smad3 phosphorylation was visibly increased under TGFβ1 stimulation. The expression of NIT1 was markedly increased following the activation of Smad3 in CRC cells (Fig. 6f).

## Discussion

CRC is a worldwide digestive tract malignant tumour. The initiation and development of CRC is a complex process involving multiple genetic changes and epigenetic modifications. In this study, we demonstrated that NIT1 is frequently downregulated in CRC and is associated with a poor survival in patients, indicating that the downregulation of NIT1 could serve as an independent prognostic marker to identify patients with poor clinical outcome. Similar to our results, the expression of NIT1 is reduced or lost in 50% of oesophageal adenocarcinoma^30^. However, whether NIT1 can be used as a universal biomarker or prognostic predictor for neoplasm needs further investigation.

We demonstrated that NIT1 suppresses CRC cell proliferation in vitro and tumour growth in vivo. NIT1-defective mouse kidney cells possess high proliferative ability^23^. The deficiency of NIT1 led to an increase in the incidence of *N*-nitrosomethylbenzylamine-induced murine gastric cancer^23^. However, a recent study has shown that NIT1 was overexpressed in human lung cancer tissues compared with that in non-malignant lung tissues and accelerated lung tumour progression in vitro and in vivo^31^. This inconsistent result may be due to the heterogeneity of the tumours. Cancer is a heterogeneous disease. In the development and progression of carcinoma, tumour cells show phenotypic and functional heterogeneity, which may result in differentially expressed genes in different types of tumours or different regions of the same tumour^32,33^.

The underlying molecular mechanisms of NIT1 in cell proliferation inhibition remain poorly identified. Here, we showed that the molecular mechanisms might involve the deceleration of the G1–S transition, upregulation of p21 and p27 and downregulation of Cyclin D1 and myc under enforced expression of NIT1. On the other hand, NIT1 induced cell apoptosis by activating caspase3 activity. Furthermore, we showed that NIT1 recruited Smad2/3 and then activated the TGFβ–Smad2/3 pathway by interacting with SARA and SMAD2/3. Previous studies have shown that the TGFβ–Smad signalling pathway plays a crucial role in many fundamental cellular processes such as cell proliferation, survival, differentiation, apoptosis and migration in numerous biological systems and is relevant to many cancer types, including CRC^9,34–36^. In other words, NIT1 activates the TGFβ–Smad2/3 pathway in CRC, exploring a novel mechanism for cell growth inhibition mediated by NIT1.

Bioinformatics analysis suggested that a predicted high-score candidate transcription factor, SMAD3, may control the transcription regulation of NIT1. Our data confirmed that SMAD3 directly binds to specific promoter regions of NIT1 and enhances the transcription of NIT1. The expression levels of SMAD3 were downregulated in CRC samples compared with their matched adjacent normal tissues and were positively correlated with NIT1 in expression. The low expression levels of SMAD3 led to a decrease in the transcriptional activation of NIT1. Recent data have indicated that Smad3 functions as a tumour suppressor by inhibiting cell proliferation and promoting apoptosis^37,38^. In this regard, NIT1-inhibited cell proliferation may be due to the transcriptional regulation of SMAD3.

**Fig. 7:**
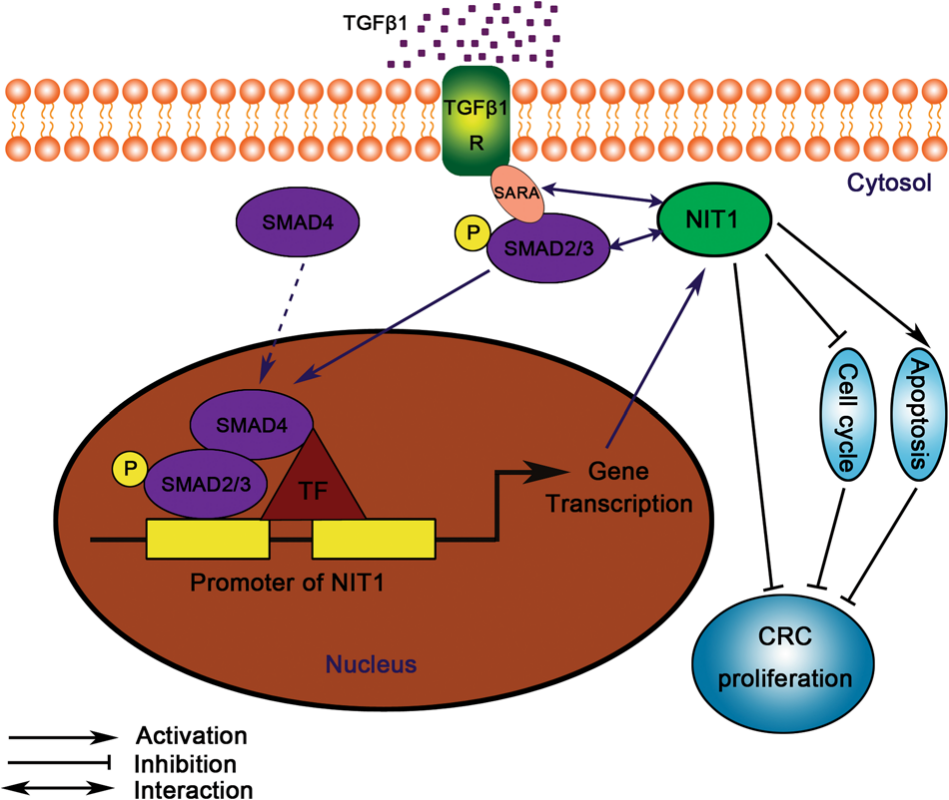
A model of NIT1 involvement in CRC. NIT1 activates the TGFβ-Smad2/3 signalling pathway by interacting with SARA and SMAD3. In addition, the induction of TGFβ1 or overexpression of SMAD3 increases the expression levels of NIT1. We demonstrated that there is a positive feedback loop involving the regulation of CRC cell proliferation between NIT1 and activation of the TGFβ–Smad pathway. We also presented that NIT1 induces cell cycle arrest and apoptosis, which also contribute to the growth inhibition of CRC

In summary, NIT1 activates the TGFβ–Smad2/3 signalling pathway, which plays a key role in the suppression of CRC proliferation by interacting with SARA. In addition, the induction of TGFβ1 or overexpression of SMAD3 increased the expression levels of NIT1. Therefore, there was a positive feedback loop involving the regulation of CRC cell proliferation between NIT1 and activation of the TGFβ–Smad signalling pathway. The increased expression of NIT1 leads to the continuous activation of the TGFβ–Smad signalling pathway and then suppresses the proliferation of CRC (Fig. 7). This outcome helps us to further comprehend the potential molecular mechanisms of CRC proliferation.

## Materials and methods

### Clinical specimens

All human CRC specimens were collected at Nanfang Hospital, Southern Medical University (Guangzhou, China) from 2012 to 2016, with the informed consent of patients, including formalin-fixed paraffin-embedded human colorectal carcinoma specimens (*n* = 69) and fresh surgically removed CRC tissues (*n* = 8). The eight cases of fresh surgically resected CRC tissues and paired adjacent normal tissues were immediately frozen in liquid nitrogen and were stored at −80 °C until further use. The use of clinical materials for research purposes has been approved by the Southern Medical University Institutional Board (Guangzhou, China).

### Cell culture

The human CRC cell lines SW620, SW480, HCT116, Lovo, HT29, LS174T, RKO, CACO2 and HEK293T were obtained from the American Type Culture Collection (ATCC, Manassas, VA, USA). SW620, SW480, HCT116, Lovo, HT29, LS174T, RKO and CACO2 were cultured under standard conditions containing RPMI1640 medium (Gibco, Grand Island, NY, USA) supplemented with 10% foetal bovine serum (FBS) (Gibco, Grand Island, NY, USA) in 5% CO_2_ at 37 °C. HEK293T cells were cultured in DMEM medium (Gibco, Grand Island, NY, USA) with 10% FBS (Gibco, Grand Island, NY, USA) at 37 °C in a humidified atmosphere with 5% CO_2_.

### RNA extraction and qRT-PCR

RNA extraction and qRT-PCR were performed as previously described using the ABI PRISM 7500 Sequence Detection System (Applied Biosystems)^39^. The results were normalized to GAPDH expression. The primer sequences for qRT-PCR are listed in Supplementary Table 1.

### Immunohistochemistry

Immunohistochemistry (IHC) staining and scoring were based on previous methods^40^. Antibodies specific to NIT1 (1:50 dilution; Proteintech, USA) and Ki67 (1:200 dilution; zsgb-bio, Beijing) were applied according to the manufacturer’s protocol.

### Western blot assay

Proteins were extracted using a lysis buffer and then were quantified using a bicinchoninic acid protein quantification kit (KeyGEN, Nanjing, China). Cell lysates were separated using 8–12% SDS-PAGE and were transferred onto a polyvinylidene difluoride membrane (Millipore, Temecula, CA, USA). The membrane was then blocked in PBST solution containing 5% non-fat milk and was incubated at 4 °C overnight with the specific primary antibodies anti-NIT1 (1:500 dilution; Proteintech, USA), anti-β-tubulin (1:2000 dilution; zsgb-bio, Beijing), anti-GAPDH (1:5000 dilution; Proteintech, USA), and anti-p53 (1:500 dilution; Proteintech, USA), anti-p21 (1:1000 dilution; Abcam, USA), anti-p27 (1:1000 dilution; Abcam, USA), anti-cyclinD1 (1:1000 dilution; Abcam, USA), anti-CDK4 (1:500 dilution; Proteintech, USA), anti-CDK6 (1:500 dilution; Proteintech, USA), anti-myc (1:500 dilution; Proteintech, USA), anti-bax (1:1000 dilution; Abcam, USA), anti-bcl2 (1:1000 dilution; Abcam, USA), anti-caspase3 (1:1000 dilution; CST, USA), anti-PARP (1:1000 dilution; CST, USA), anti-SARA (1:500 dilution; Abcam, USA), anti-Smad2/3 (1:500 dilution; Abcam, USA), anti-Smad2 (1:1000 dilution; Abcam, USA), anti-Smad3 (1:1000 dilution; Abcam, USA), anti-p-Smad2 (1:500 dilution; CST, USA), and anti-p-Smad3 (1:1000 dilution; CST, USA), followed by incubation with their respective appropriate second antibodies. The bands were detected using the Pierce ECL Western Blotting Substrate (Thermo Scientific, USA).

### Transfection and lentiviral transduction

Lentiviral constructs expressing NIT1 were purchased from GeneCopoeia (USA) and were used to infect CRC cells to establish CRC cells constitutively expressing NIT1. Lentiviral constructs repressing NIT1 were purchased from GenePharma (Suzhou, China) and were used to establish CRC cell lines constitutively repressing NIT1. The stable cell lines were selected with 2 μg/mL puromycin. The shRNA or siRNA used to inhibit the expression of NIT1 or SARA was purchased from GenePharma (Suzhou, China) (for details, see Supplementary Table 2). The SMAD3 expression plasmid was purchased from GeneCopoeia (USA). Cells were transfected with siRNA oligonucleotides or plasmids using Lipofectamine 2000 (Invitrogen, USA) or Lipofectamine 3000 (Invitrogen, USA).

### CCK-8 cell proliferation assay and colony formation assay

The CCK-8 cell proliferation assay and colony formation assay were performed as previously described^40^.

### Tumourigenesis in nude mice

Male 4 to 6-week-old athymic BALB/c nude mice (nu/nu) were purchased from the Animal Center of Guangdong Province (Guangzhou, China) and were maintained in laminar flow cabinets under specific pathogen-free conditions. All animal experiments were carried out in accordance with animal protocols approved by the Animal Care and Use Committee of Southern Medical University. In total, 1*10^7^ cells were injected into the subcutaneous tissues of nude mice (*n* = 5 per group). The tumour size was measured using a slide caliper, and the tumour volume was calculated by the formula (*V* = 1/2*length*width*height). After 15 days, the tumours were excised and fixed with 10% neutral-buffered formalin, and then 4-μm sections were prepared. The sections were stained with haematoxylin–eosin (HE) according to standard protocols and then were further subjected to IHC staining using anti-Ki67.

### Flow cytometry

Flow cytometry assays were performed as previously described^41^. The Cell Cycle Detection Kit (KeyGEN, Nanjing, China) and Annexin V-APC/7-AAD Apoptosis Detection Kit (KeyGEN, Nanjing, China) were used according to the manufacturer’s instruction.

### Co-immunoprecipitation

Proteins were extracted from CACO2 cells with lysis buffer. NIT1 (Abcam, USA), SARA (Abcam, USA) or SMAD2/3 (Abcam, USA) antibody was added to the cell lysates, followed by incubation overnight at 4 °C. The following day, ~30 μl of magnetic beads was added. The beads were incubated for 6 h and were washed six times in PBS, and then the proteins were eluted in Laemmli buffer. Western blotting was used to analyse the interacting proteins.

### Luciferase reporter assay

The pGL3-WT luciferase reporter plasmid was obtained by ligating oligonucleotides containing a WT NIT1 promoter into the pGL3-Basic vector (Promega, USA). The pGL3-Mut plasmid with a mutant target site in the E-box was synthesized by Ruibiotech (Beijing, China). Cells were seeded in 24-well plates (1*10^5^ per well) and were cultured for 24 h before transfection. The pGL3-Basic, pGL3-WT or pGL3-MUT plasmid was co-transfected with the SMAD3 plasmid (GeneCopoeia, USA) and pRL-TK plasmid (Promega, USA) in HEK293T and SW620 cells using Lipofectamine 3000 (Invitrogen, USA). The pRL-TK plasmid vectors were used as controls. Luciferase activity was measured using the Dual-Luciferase Reporter Assay System (E1910; Promega, USA) after 72 h.

### Chromatin immunoprecipitation

ChIP assays were performed using the EZ-CHIP kit (Millipore, Temecula, CA, USA). In total, 1 × 10^6^ cells were cultured and lysed, and then chromatin lengths ranging from 200 to 1000 bp were produced by sonication. Anti-SMAD3 (Abcam, USA) or anti-IgG (Santa Cruz, USA) antibody was used to precipitate the DNA–protein complexes overnight at 4 °C. Protein–DNA complexes were recovered using magnetic beads, washed, and then eluted. Cross-links were reversed at 65 °C overnight. The immunoprecipitated DNA was examined by PCR and qRT-PCR. The primers specific for the NIT1 promoter containing the E-box were 5′-ATCTTCCCAGACCCTACAT-3′ (Forward) and 5′-CTTTACTCCAGCACCACAT-3′ (Reverse).

### Statistical analysis

SPSS 20.0 software (IBM) was used for all statistical analyses. The data were presented as means ± SD in at least three independent experiments. The differences among groups were tested using one-way ANOVA or two-tailed Student’s *t*-test. Relationships between NIT1 expression and clinicopathologic characteristics were analysed by *χ*^2^ test. The survival curve was plotted by the Kaplan–Meier method and was compared using the log-rank test. Spearman’s correlation coefficient was used to examine the linear relationship between the expression levels of NIT1 and SMAD3 in CRC tissues. *p* < 0.05 was considered statistically significant: *p < 0.05; **p < 0.01; ***p < 0.001.

### Accession numbers for data sets

The clinical data sets generated and reanalysed in the study were obtained from the GEO database (GSE24551)^42,43^.

## Electronic supplementary material

Supplementary Figure

Supplementary Table

Supplementary Summary
