# Supplementary material for: NIT1 suppresses tumour proliferation by activating the TGFβ1–Smad2/3 signalling pathway in colorectal cancer
Source: Cell Death Dis. 2018 Feb 15;9(3):263. doi: 10.1038/s41419-018-0333-3 (PMC5833788; doi:10.1038/s41419-018-0333-3)
Supplement: Supplementary file 1 — Supplementary Figure [file 41419_2018_333_MOESM1_ESM.pdf]

Supplementary Figure

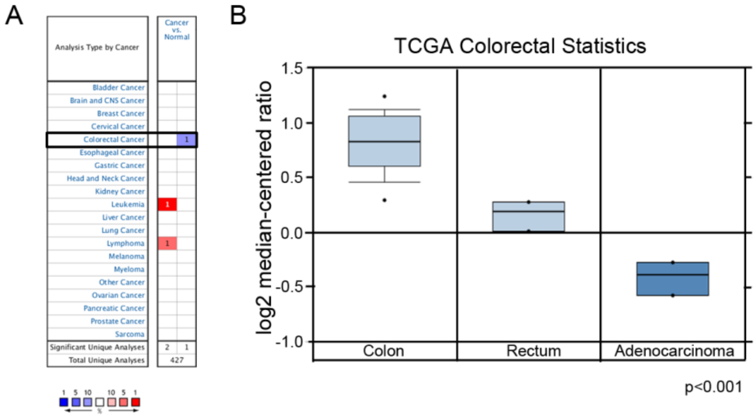

Supplementary Figure S1: The expression of NIT1, data from Onconmine database

(A) Analysis of NIT1 expression in various types of malignancies. The red indicates upregulation, and the blue represents downregulation. (B) Analysis of NIT1 expression in TCGA colorectal large sample chip database (n=237 samples).

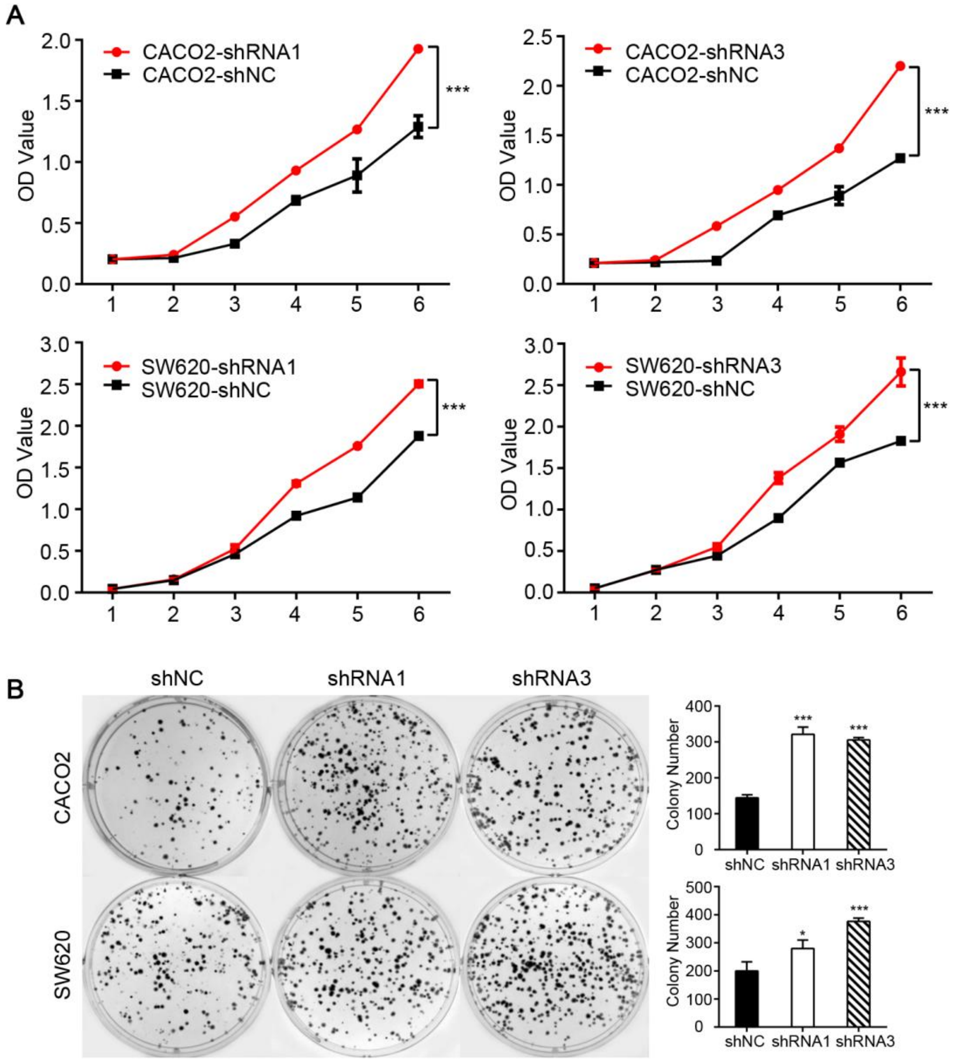

Supplementary Figure S2: Knockdown of NIT1 promotes CRC cell proliferation in vitro

(A) Downregulation of NIT1 promotes CRC cell proliferation by CCK-8 cell proliferation assays. The data are represented as the means ± SD of 5 samples. (B) Downregulation of NIT1 promotes CRC cell proliferation by colony formation assays. The data are represented as the means ± SD of 3 samples.

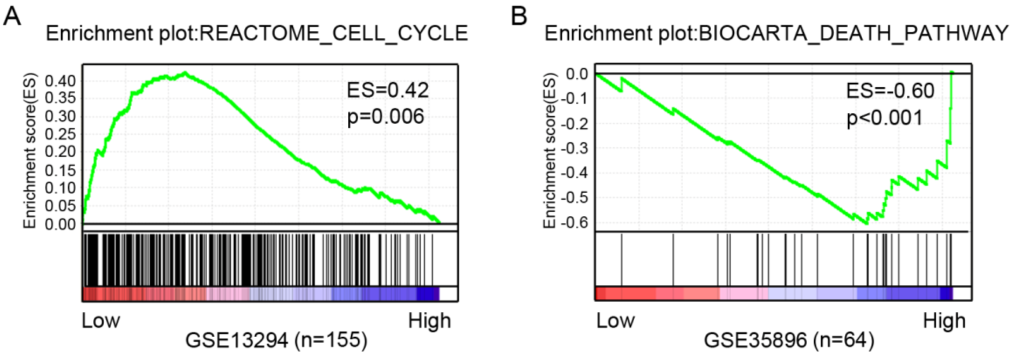

Supplementary Figure S3: GSEA (Gene Set Enrichment Analysis)

(A)The enrichment of cell cycle-related pathway in low NIT1 expression group (GSE13294). (B) The enrichment of apoptosis-related pathway in high NIT1 expression group (GSE=35896).

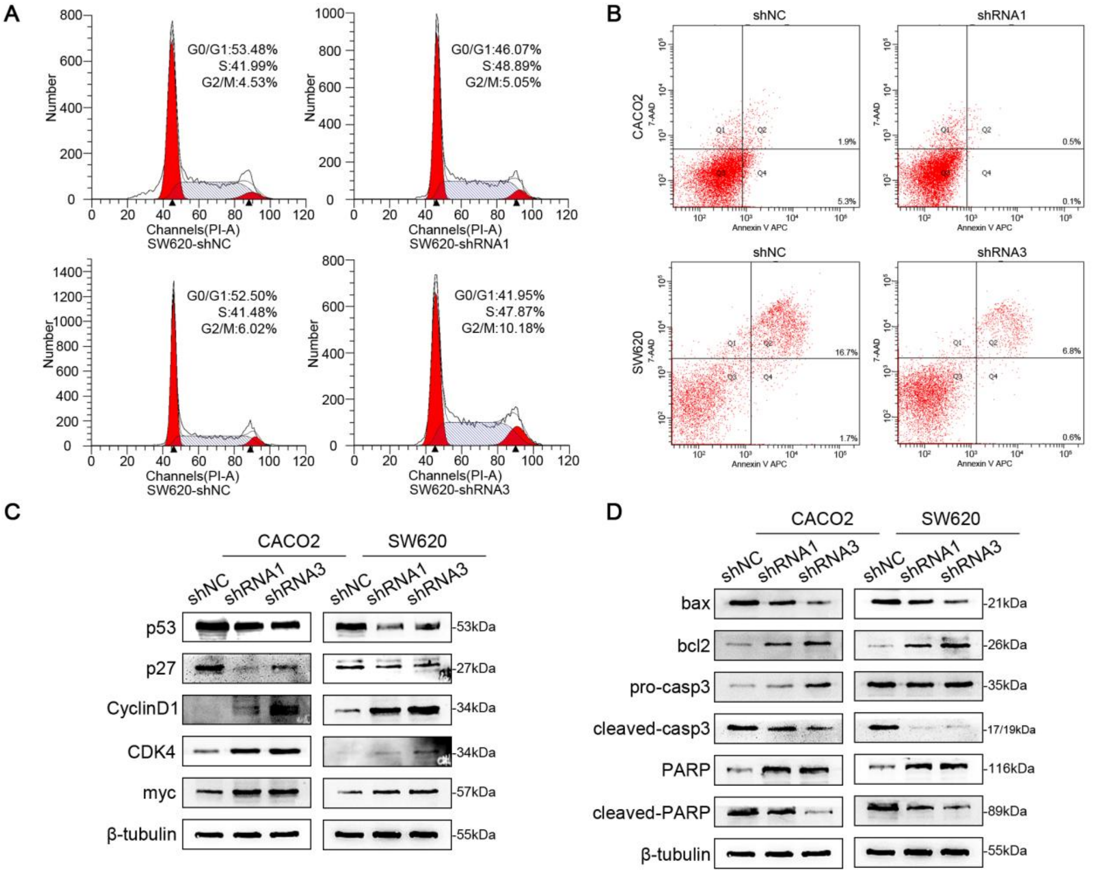

Supplementary Figure S4: NIT1 induces cell cycle arrest and apoptosis

(A) The cell cycle distribution was analysed after the knock down of NIT1 by flow cytometry in CRC cells. (B) Downregulation of NIT1 induced a decrease in the total apoptosis rate as analysed by flow cytometry in CRC cells. (C) The expression levels of cell cycle regulators, including p53, p27, CyclinD1, CDK4, and myc, were analysed by Western blot assays. (D) The expression of apoptosis-associated proteins, including bax, bcl2, caspase3 and PARP, were analysed by Western blot assays.

Model ID	Model name	Score	Relative score	Start	End	Strand	predicted site sequence
MA0513.1	SMAD2::SMAD3::SMAD4	7.349	0.804819372612081	350	362	-1	CTGCCTCCCAGGT
MA0513.1	SMAD2::SMAD3::SMAD4	7.099	0.80073924221586	363	375	-1	CTGACTGCAACCT
MA0513.1	SMAD2::SMAD3::SMAD4	8.740	0.827521218136654	879	891	1	GTGGCTCACGCCT
MA0513.1	SMAD2::SMAD3::SMAD4	14.073	0.914558559748839	1678	1690	1	GTGGCTCTCACCT
MA0513.1	SMAD2::SMAD3::SMAD4	7.262	0.803399487234196	1752	1764	-1	GTGCGTGGCACCT

Supplementary Figure S5: Bioinformatics analysis the transcription factors binding to the NIT1 promoter (JASPAR)

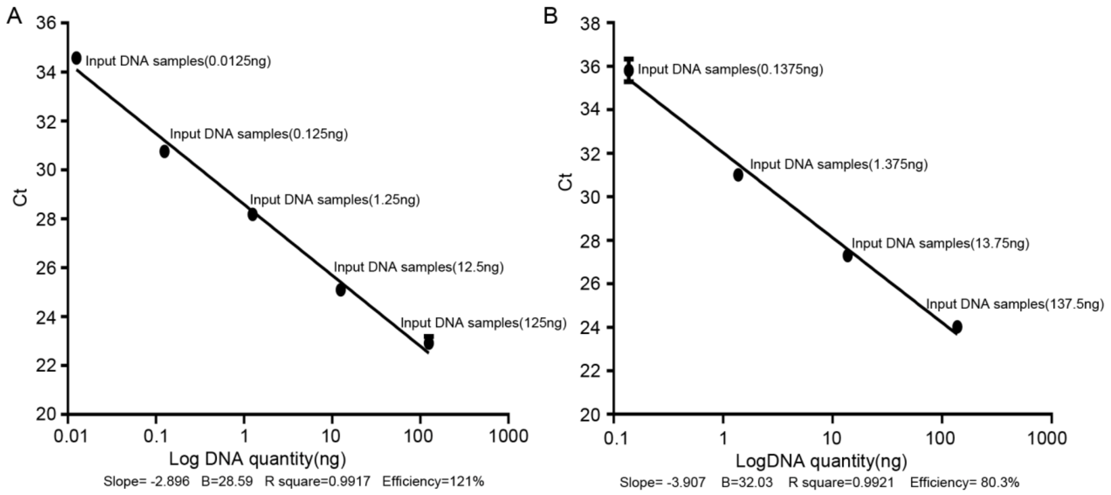

Supplementary Figure S6: Standrad curve produced from ten-fold dilutions of Input DNA

Five (A) or four (B) ten-fold dilutions of Input DNA were qPCR amplified along with the Chip and IgG samples using primer specific to the NIT1 promoter in CACO2 cells (A) or RKO cells (B). The values for each amount of Input DNAwere plotted and used to produce the standard curve. Its slope and y-intercept values were used with the Ct values of the samples to calculate the fold enrichment.
