# Supplementary material for: NIT1 suppresses tumour proliferation by activating the TGFβ1–Smad2/3 signalling pathway in colorectal cancer
Source: Cell Death Dis. 2018 Feb 15;9(3):263. doi: 10.1038/s41419-018-0333-3 (PMC5833788; doi:10.1038/s41419-018-0333-3)
Supplement: Supplementary file 2 — Supplementary Table [file 41419_2018_333_MOESM2_ESM.pdf]

Supplementary Table

Supplementary Table 1: Primer sequences used for qRT-PCR (5' to 3')

Gene	Forward primer	Reverse primer
NIT1	GTGTGCCAGGTAACATCGAC	AGGGTCCCGTGCAATGAAG
SMAD3	CCATCTCCTACTACGAGCTGAA	CACTGCTGCATTCCTGTTGAC

Supplementary Table 2: Sequences used for the knockdown of genes (5' to 3')

Gene	Sense（5'-3'）	Antisense（5'-3'）
NIT1 shRNA1	GCTGTGTGCCAGGTAACAT	
NIT1 shRNA2	GGAAGAATACACCCAGCTT	
NIT1 shRNA3	GCAAGATTGGTCTAGCTGT	
NIT1 shRNA4	GCTGGAGCAGAGATACTTA	
SARA siRNA	GUGUCCUACUUCAUCUGAUTT	AUCAGAUGAAGUAGGACACTT
